# Supplementary material for: Identification of Freezing-Responsive microRNAs and Their Targets in Chinese Jujube by Small RNA and Degradome Sequencing
Source: Int J Mol Sci. 2026 Sep 4;27(17):7912. doi: 10.3390/ijms27177912 (PMC13566890; doi:10.3390/ijms27177912)
Supplement: Supplementary file 1 [file ijms-27-07912-s001.zip › Supplementary Material S2/novel_miR_54.pdf]

Provisional ID : novel_miR_54
Score total : 96155
Score for star read(s) : 3.9
Score for read counts : 96149.1
Score for mfe : 0.5
Score for randfold : 1.6
Score for cons. seed :
Total read count : 188604
Mature read count : 187966
Loop read count : 0
Star read count : 638

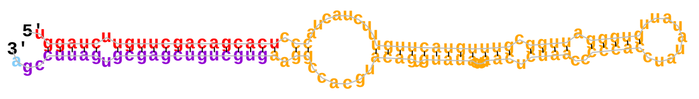

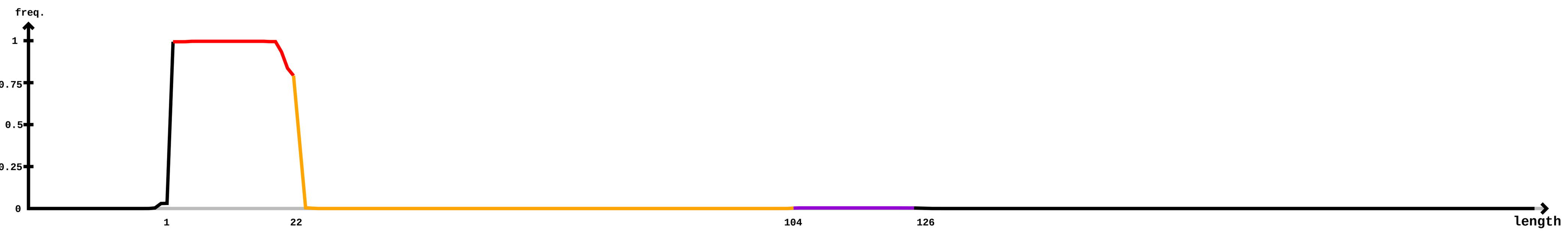


```
                         Mature                                                                                                Star
5'- gggugguuguucauuuuacccucuggaucuuguucgacagcacucccaucaucuuuguucauguuugcgguuagggugguuauauauccacccccaaucucaaucucauaugugacaugcaccggaagugcugucgagcgugauuccgaaggugcuguucagcaacauacagauggaucgagcaacuacacugccugcuauauaugcccaucaucaucuucuacuuaaccuugcuuauuuauuuauuauuca -3'  obs
    gggugguuguucauuuuacccucuggaucuuguucgacagcacucccaucaucuuuguucauguuugcgguuagggugguuauauauccacccccaaucucaaucucauaugugacaugcaccggaagugcugucgagcgugauuccgaaggugcuguucagcaacauacagauggaucgagcaacuacacugccugcuauauaugcccaucaucaucuucuacuuaaccuugcuuauuuauuuauuauuca      exp
    ..((((((((.....((((.((.(((((.((((((((((((((.((........(((((((((((.((((.(((((.........)))))..)))).)))....))))).))))......)).)))))))))))))).))))).)).))))......))))))))...(((((....((((...........))))........)))))......................................  reads  mm  sample
    ............uuuacccucuggaucuuguA...                1    1   JCK-1
    ............uuuacccucuggaucuuguuc...               1    0   JCK-3
    ............uuuacccucuggaucuuguuc...               1    0   JCK-2
    .................ccucuggaucuuguucgaca...           1    0   JCK-1
    .................ccucuggaucuuguucgacag...          5    0   LT-3
    .................ccucuggaucuuguucgacagc...         6    0   JCK-1
    .................ccucuggaucuuguucgacagc...         4    0   JCK-3
    .................ccucuggaucuuguucgacagc...         2    0   LT-3
    .................ccucuggaucuuguucgacagc...         2    0   LT-2
    .................ccucuggaucuuguucgacagc...         2    0   LT-1
    .................ccucuggaucuuguucgacagc...         8    0   JCK-2
    .................ccucuggaucuuguucgacagcacu...      1    0   JCK-1
    .................ccucuggaucuuguucgacagcacu...      1    0   JCK-3
    .................ccucuggCucuuguucgacagcacu...      1    1   JCK-2
    .................ccucuggaucuuguucgacagcacu...      1    0   JCK-2
    ..................cucuggaucuuguucgac...            1    0   LT-3
    ..................cucuggaucuuguucgaca...           6    0   LT-3
    ..................cucuggaucuuguucgaca...           2    0   LT-1
    ..................cucuggaucuuguucgaca...           6    0   LT-2
    ..................cucuggaucuuguucgaca...           8    0   LCK-3
    ..................cucuggCucuuguucgacagc...         1    1   JCK-2
    ..................cucuggaucuuguucgacagc...         5    0   LCK-3
    ..................cucuggaucuuguucgacagc...         8    0   LT-2
    ..................cucuggaucuuguucgacagc...         43   0   JCK-2
    ..................cucuggaucuuguucgacagc...         20   0   LT-1
    ..................cucuggaucuuguucgacagc...         22   0   LT-3
    ..................cCcuggaucuuguucgacagc...         2    1   LT-1
    ..................cucuggaucuuguucgacagc...         34   0   JCK-3
    ..................cucuggaucuuguucgacagc...         36   0   JCK-1
    ..................cNcuggaucuuguucgacagca...        1    1   JCK-2
    ..................cucuggaucuuNuucgacagca...        1    1   JCK-1
    ..................cucuggaucuuguucgacagca...        137  0   JCK-2
    ..................cucuggaucuuguucgacagca...        103  0   JCK-3
```

Mature / Star			
gggguguuguucauuuuacccucuggaucuuguucgacagcacucccaucaucuuuguucauguuugcgguuaggguguuauauauccacccccaaucucaaucucauaugugacaugcaccggaagugcugucgagcgugauuccgaaggugcuguucagcaacauacagauggaucgagcaacuacacugccugcuauauaugcccaucaucaucuucuacuuaaccuugcuuauuuauuuauuauuca			
...................cucuggaucuuguucgacagca	19	0	LT-1
...................cucuggaucuuguucgaAagca	1	1	JCK-1
...................cucuggaucuuguucgacagca	24	0	LCK-3
...................cucuggaucuuguucgacaNca	1	1	JCK-1
...................cucuggaucuuguucgacagcU	1	1	JCK-1
...................cucuggaucuuguucgacagca	6	0	LT-2
...................cucuggaucuuguucgacagUa	2	1	JCK-2
...................cucuggaucuuguucgacagcG	1	1	JCK-1
...................Aucuggaucuuguucgacagca	2	1	JCK-3
...................cucuggaucuuguucgacagca	12	0	LT-3
...................cucuggaucuuguucgacagcU	3	1	JCK-2
...................cuUuggaucuuguucgacagca	1	1	JCK-3
...................cucuggaucuuguucgacagca	81	0	JCK-1
...................cucuggaucuuguucgCcagca	1	1	LT-1
...................cucuggaucuuguucgacCgca	1	1	JCK-1
...................cNcuggaucuuguucgacagca	1	1	JCK-3
...................cucuggaucuuguucgacagcacu	1	0	JCK-3
....................ucuggaucuuguucgaca	14	0	JT-2
....................ucuggaucuuguucgaca	17	0	LT-1
....................ucuggaucuuguucgacU	1	1	LT-3
....................ucuggaucuuguucgaca	12	0	JT-1
....................ucuggaucuuguucgaca	17	0	JT-3
....................ucuggaucuuguucgaca	53	0	LT-3
....................ucuggaucuuguucgaca	23	0	JCK-2
....................ucuggaucuuguucgCca	1	1	LT-3
....................ucuggaucuuguucgaca	37	0	LT-2
....................ucuggaucuuguucgaca	25	0	JCK-1
....................ucuggaucuuguucgaca	22	0	JCK-3
....................ucuggaucuuguucgacU	1	1	JCK-1
....................ucuggaucuuguucgaca	76	0	LCK-3
....................ucuggaucuuguucgacU	2	1	LCK-3
....................ucuggaucuuguucgacU	1	1	JCK-2
....................ucuggaucuuguucgacU	2	1	LT-1
....................ucuggaucuuguucgacaA	1	1	LT-1
....................ucuggaucuuguucgacag	1	0	JCK-1
....................ucuggaucuuguucgacaU	2	1	JCK-3
....................ucuggaucuuguucgacag	1	0	LT-3
....................ucuggaucuuguucgacaU	2	1	JCK-2
....................ucuggaucuuguucgacagc	56	0	JCK-2
....................ucuggaucuuguucgacagc	62	0	LCK-3
....................ucuggaucuuguucgacagU	1	1	LT-1
....................ucuggCucuuguucgacagc	1	1	LT-1
....................ucuggaucuuguucgacagc	20	0	LCK-1
....................ucuggaucuuguucgacagc	4	0	JT-3
....................ucuggaucuuguucgacagc	29	0	JCK-3
....................ucuggaucuuguucgacagc	5	0	JT-2
....................ucuggaucuuguucgacagc	28	0	LCK-2
....................ucuggaucuuguucgacagc	33	0	JCK-1
....................ucuggNucuuguucgacagc	1	1	LCK-1
....................ucuggaucuuguucgacagc	69	0	LT-2
....................ucuggaucuuguucgacagc	80	0	LT-3
....................ucuggaucuuguucgacagc	52	0	LT-1
....................ucNggaucuuguucgacagca	1	1	LCK-1
....................ucuggaucuuguucgacagca	56	0	JT-1
....................ucuggaucuuguucgCcagca	1	1	JCK-1
....................ucuggaucuuguucgacaAca	1	1	JCK-2
....................ucuggaucCuguucgacagca	1	1	JCK-2
....................ucuggaucuuguucAacagca	1	1	JCK-1
....................ucuggaucuuguucgacagca	19	0	JT-3
....................ucuggaucuugNucgacagca	2	1	JCK-1
....................ucuggaucuuguucgaUagca	1	1	JCK-3
....................ucuggaucuuguucgaAagca	1	1	JCK-1
....................ucuggaucuuguucgacagca	83	0	LT-2
....................ucuggaucuuguucgacagcU	8	1	JCK-2
....................ucuggaucuuguucgaAagca	1	1	JCK-3
....................ucuggaucuuguucgacagcU	3	1	LCK-3
....................ucuggaucuuguucgacaUca	3	1	JCK-2
....................ucuggaucuuguucgacagcU	2	1	JCK-1
....................ucuggauAuuguucgacagca	1	1	JCK-3
....................ucuggaucuuguucgacagcU	3	1	LT-2

Mature (red)   **Star** (purple)

ggguguuguucauuuuacccucuggaucuuguucgacagcacucccaucaucuuuguucauguuugcgguuagggguguuauauauccacccccaaucucaaucucauaugugacaugcaccggaagugcugucgagcgugauuccgaaggugcuguucagcaacauacagauggaucgagcaacuacacugccugcuauauaugcccaucaucaucuucuacuuaaccuugcuuauuuauuuauuauuca

....................ucuggaucuuguucgacagca	31	0	LCK-2
....................ucuggaucuuguucgacagca	9	0	JT-2
....................ucuggaucuuguucgacaUca	1	1	JCK-1
....................ucuggaucuuguucgacagca	27	0	LCK-1
....................ucuggaucuuguucgacagcC	1	1	JCK-1
....................ucugAaucuuguucgacagca	1	1	JCK-1
....................ucuggaucuuguucgacagca	882	0	JCK-1
....................ucuggaucuuguucgCcagca	1	1	JT-1
....................ucuggaucuuguucgacagca	128	0	LT-3
....................ucuggCucuuguucgacagca	4	1	JCK-2
....................ucuggaucuuguucgacagca	76	0	LCK-3
....................ucuggCucuuguucgacagca	1	1	JCK-3
....................Ccuggaucuuguucgacagca	1	1	LT-3
....................ucuggaucuuguucgacagcU	2	1	LT-3
....................ucuggaucuuguucgacagcU	3	1	JCK-3
....................ucuggaucuuguucgacagcN	1	1	JCK-1
....................ucuggaucCuguucgacagca	1	1	LT-2
....................ucuggaucuuguucgacagca	109	0	LT-1
....................ucuggaucuuguucgacagca	1162	0	JCK-3
....................ucuggaucuuguucgCcagca	1	1	LT-1
....................uUuggaucuuguucgacagca	5	1	JCK-3
....................ucCggaucuuguucgacagca	1	1	JCK-3
....................ucuggaucuuguucgacagca	1463	0	JCK-2
....................uUuggaucuuguucgacagca	5	1	JCK-1
....................Ccuggaucuuguucgacagca	1	1	JCK-2
....................uNuggaucuuguucgacagca	2	1	JCK-2
....................ucuggaucuuguucgaAagca	1	1	JCK-2
....................uUuggaucuuguucgacagca	5	1	JCK-2
....................ucuggaucuuguucgacagUa	1	1	JCK-3
....................ucuggaucuuguucgacagcac	11	0	LT-3
....................ucuggaucuuguucgacagcaA	1	1	JCK-3
....................ucuggaucuuguucgacagcac	37	0	JCK-2
....................ucuggaucuuguucgacagcac	16	0	JCK-1
....................ucuggaucuuguucgacagcac	10	0	LT-1
....................ucuggaucuuguucgacagcac	30	0	JCK-3
....................ucuggaucuuguucgacagcac	16	0	LCK-3
....................ucuggaucuuguucgacagcac	7	0	LT-2
....................ucuggaucuuguucgacagcacu	9	0	JCK-2
....................ucuggaucuuguucgacagcacu	8	0	LCK-3
....................ucuggaucuuguucgacagcacA	1	1	JCK-2
....................ucuggaucuuguucgacagcacC	1	1	LT-3
....................ucuggaucuuguucgacagcaUu	1	1	LT-3
....................ucuggaucuuguucgacagcacu	2	0	LT-2
....................ucuggaucuuguucgacagcacA	1	1	JCK-3
....................ucuggaucuuguucgacagcacu	4	0	JCK-1
....................ucuggaucuuguucgacagcacA	2	1	JCK-1
....................ucuggaucuuguucgacagcacu	3	0	LT-3
....................ucuggaucuuguucgacagcaAu	3	1	LT-3
....................ucuggaucuuguucgacagcacu	5	0	JCK-3
....................ucuggaucuuguucgacagcacuc	1	0	JCK-3
....................ucuggaucuuguucgacagcacuc	3	0	LT-1
....................ucuggaucuuguucgacagcacuc	3	0	JCK-2
....................ucuggaucuuguucgacagcacuc	5	0	JCK-1
....................ucuggaucuuguucgacagcacuc	1	0	LT-3
....................ucuggaucuuguucgacagcacuU	1	1	LT-1
.....................cuggaucuuguucgacagc	4	0	LT-3
.....................cuggaucuuguucgacagc	3	0	LT-2
.....................cuggaucuuguucgacagc	1	0	LT-1
.....................cuggaucuuguucgacagc	1	0	LCK-3
.....................cuggaucuuguucgacagca	15	0	JCK-1
.....................cuggaucuuguucgacagca	4	0	LT-3
.....................cuggaucuuguucgacagca	17	0	JCK-2
.....................cuggaucuuguucgacagcU	1	1	JCK-3
.....................cuggaucuuguucgacagca	2	0	LT-1
.....................cuggaucuuguucgacagca	12	0	JCK-3
.....................cuggaucAuguucgacagca	1	1	LT-1
.....................cuggaucuuguucgacagca	7	0	LCK-3
.....................cuggaucuuguucgacagcac	1	0	JCK-3
.....................cuggaucuuguucgacagcac	4	0	LT-2
.....................cuggaucuuguucgacagcac	2	0	JCK-2

Mature   Star

gguguuguucauuuuacccucuggaucuuguucgacagcacucccaucaucuuuguucauguuugcgguuagggugguuauauauccacccccaaucucaaucucauaugugacaugcaccggaagugcugucgagcgugauuccgaaggugcuguucagcaacauacagauggaucgagcaacuacacugccugcuauauaugcccaucaucaucuucuacuuaaccuugcuuauuuauuuauuauuca

cuggaucuuguucgacagcacu	2	0	LT-1
Uuggaucuuguucgacagcacu	1	1	JCK-3
cuggaucuuguucgacagcacu	5	0	LCK-3
cuggaucuuguucgacagcacu	13	0	JCK-3
cuggaucuuguucgacagcacu	15	0	JCK-1
cuggaucuuguucgacagcacu	3	0	LT-2
cuggaucuuguucgacagcacu	2	0	LT-3
cuggaucuuguucgacagcacu	27	0	JCK-2
cuggaucuuguucgacagcacuc	1	0	JCK-1
cuggaucuuguucgacagcacuc	6	0	JCK-2
cuggaucuuguucgacagcacuc	1	0	JCK-3
cuggaucuuguucgacagcacuc	2	0	LT-3
Uuggaucuuguucgacagcacuc	1	1	JCK-2
uggaucuuguucgacagc	81	0	JT-1
uggaucuuguucgCcagc	2	1	LCK-2
uggaucuuguucgacGgc	1	1	JCK-2
uggaucuuguuNgacagc	1	1	JCK-2
uggaucuuguucgacagU	3	1	LT-1
ugAaucuuguucgacagc	2	1	JCK-2
uggaucuuguucgCcagc	2	1	JCK-3
uggaucuuguucgaUagc	1	1	LCK-3
uggaucuuguucgacagU	5	1	LCK-3
uggaucuuguucgCcagc	1	1	LT-3
uNgaucuuguucgacagc	1	1	LCK-1
ugAaucuuguucgacagc	1	1	LCK-3
uggaucuuguucgacagc	74	0	JT-3
uUgaucuuguucgacagc	1	1	LT-3
uAgaucuuguucgacagc	2	1	JCK-2
uggaucuuguucgNcagc	2	1	LCK-1
uggaucuuguucgacaUc	1	1	JCK-1
uggaucuuguucgacagc	454	0	JCK-1
uggaucuuguucgacagA	1	1	LT-3
ugUaucuuguucgacagc	1	1	LT-3
Cggaucuuguucgacagc	1	1	LT-2
uggaucuuguuNgacagc	1	1	JT-1
ugAaucuuguucgacagc	1	1	JCK-3
uggaucuuguucgacagU	1	1	LT-2
uggaucuuguucgacCgc	1	1	JCK-3
uggaucuuguucAacagc	1	1	LT-2
uggCucuuguucgacagc	1	1	JCK-1
uggaCcuuguucgacagc	4	1	LCK-3
uAgaucuuguucgacagc	1	1	LT-2
uggaucuuguucgCcagc	8	1	LT-2
uggaucuuguucgaAagc	1	1	LT-2
uggaucuuguucgacaAc	1	1	LT-1
ugNaucuuguucgacagc	4	1	LT-2
uggGucuuguucgacagc	1	1	JT-1
uggCucuuguucgacagc	1	1	LCK-1
uggaucuuguucgCcagc	1	1	LCK-1
uggaucuuguucgacaUc	6	1	LT-2
uggaucuuguucgacagc	3342	0	LCK-3
uggaucuuguucgacagU	3	1	JCK-3
uggaucuuguuNgacagc	1	1	JCK-1
uggaucuuguucgacagc	663	0	LCK-2
ugNaucuuguucgacagc	1	1	LCK-3
uggCucuuguucgacagc	1	1	LCK-2
uggaucuuguucgacagc	48	0	JT-2
uggCucuuguucgacagc	3	1	LT-1
uggaucuuguucgacagc	659	0	LCK-1
uggaucuuguucgCcagc	3	1	JCK-2
ugAaucuuguucgacagc	2	1	LT-2
uggaucuuguucgacagc	890	0	LT-1
uggCucuuguucgacagc	3	1	LT-3
uNgaucuuguucgacagc	1	1	JT-1
uggaucuuguucgacagc	1173	0	LT-3
uNgaucuuguucgacagc	1	1	JCK-2
uggaucuuguucUacagc	1	1	JCK-3
uggGucuuguucgacagc	2	1	JCK-1
uggCucuuguucgacagc	3	1	JCK-3
uggaucuuguucgacagc	616	0	JCK-3

Mature **Star**

gggguguuguucauuuuacccucuggaucuuguucgacagcacucccaucaucuuuguucauguuugcgguuagggguguuauauauccacccccaaucucaaucucauaugugacaugcaccggaagugcugucgagcgugauuccgaaggugcuguucagcaacauacagauggaucgagcaacuacacugccugcuauauaugcccaucaucaucuucuacuuaaccuugcuuauuuauuuauuauuca

uggaucuuguucgGcagc	1	1	LCK-3
ugNaucuuguucgacagc	1	1	LCK-1
uggaucuuguucgacagU	1	1	JCK-1
uggaucuuguucgaUagc	1	1	JCK-1
uggaucuuguucgacagU	2	1	JCK-2
uggaucuuguucgacaUc	2	1	JCK-3
uggaucuuguucgacCgc	1	1	LT-2
uAgaucuuguucgacagc	1	1	LCK-1
uggCucuuguucgacagc	6	1	LCK-3
uAgaucuuguucgacagc	2	1	LCK-3
uggaucuuguucgacagU	15	1	LT-3
uggaucuuguucgacaAc	1	1	LT-2
uggaucuuguucgCcagc	2	1	LT-1
uggaucuuguucgaUagc	1	1	LT-2
uggaucuuguucgaAagc	1	1	LCK-3
uggaucuuguucgacagc	2025	0	LT-2
ugAaucuuguucgacagc	1	1	LCK-1
uggaucuuguucgacaAc	1	1	LCK-3
uggaucuuguucgacagc	794	0	JCK-2
uNgaucuuguucgacagc	1	1	LT-3
uggCucuuguucgacagc	19	1	LT-2
uggaucuuguucgCcagc	4	1	LCK-3
uggaucuuguucgacagUa	5	1	LT-1
uggaucuuguucgacGgca	1	1	JT-1
uggCucuuguucgacagca	8	1	LCK-2
uggauNuuguucgacagca	3	1	LCK-1
uggaucuuguucgacagcC	2	1	LT-3
uggaucuuguucgaGagca	1	1	LCK-1
uggaucuuguucgacagca	496	0	LT-1
uNgaucuuguucgacagca	3	1	JT-1
uggaucuuguucgacagca	1113	0	LT-3
uggaucuuguucgacagcU	164	1	LT-3
uggNucuuguucgacagca	1	1	JCK-1
uggGucuuguucgacagca	1	1	LCK-3
uggaucCuguucgacagca	1	1	LCK-1
uggaucuuguucgCcagca	2	1	LT-1
uggaucuuguucgacagUa	5	1	LT-3
uggaucuuguucgacagca	932	0	JCK-2
uggaucuuguucAacagca	1	1	LT-3
uggaucuuAuucgacagca	1	1	LCK-1
uggaucuuguucgacCgca	2	1	LCK-2
uggaucuuguucgacagcU	194	1	JCK-2
uggaucuuguucgacCgca	2	1	LT-2
uggaucuuguucgCcagca	5	1	LCK-1
uggaucuuguucgacagca	294	0	JT-3
uggaucuuguucgacagUa	10	1	JT-1
uAgaucuuguucgacagca	1	1	LT-2
uggCucuuguucgacagca	2	1	LCK-1
uggaucuuguucgacCgca	2	1	JCK-2
uggaucuuguucgacagcC	1	1	JCK-2
Cggaucuuguucgacagca	1	1	LT-2
uggaucuuguuNgacagca	1	1	JT-2
uggaucuuguucgacagUa	4	1	JCK-3
uggaucuuguucgCcagca	1	1	LCK-2
uggGucuuguucgacagca	1	1	LCK-2
uggaucuuguucgacaUca	1	1	LT-3
uUgaucuuguucgacagca	1	1	LCK-2
uggaucuuguucgacagca	575	0	JT-1
uNgaucuuguucgacagca	3	1	JCK-2
uggaucuuguucgacagGa	1	1	JCK-1
uggaucuuguucgacagcG	3	1	JCK-2
uggaucuuguucgaAagca	1	1	LT-3
uggCucuuguucgacagca	5	1	LT-2
uggaucuuguucgaAagca	3	1	LCK-2
uggCucuuguucgacagca	1	1	JCK-1
uggaucuuguucgacagUa	11	1	LT-2
ugCaucuuguucgacagca	1	1	LT-2
uggaucuuguucgacagca	1924	0	LCK-1
uggaucuuguucgaUagca	1	1	LT-2
uggCucuuguucgacagca	5	1	LT-1

Mature Star

gggguguuguucauuuuacccucuggaucuuguucgacagcacucccaucaucuuuguucauguuugcgguuagggugguuauauauccacccccaaucucaaucucauaugugacaugcaccggaagugcugucgagcgugauuccgaaggugcuguucagcaacauacagauggaucgagcaacuacacugccugcuauauaugcccaucaucaucuucuacuuaaccuugcuuauuuauuuauuauuca

.....................uggaucuuguucgacaUca..............................	1	1	JCK-3
.....................uggaucuuguucgacagUa..............................	1	1	JCK-1
.....................uggaucuuguucgacagUa..............................	9	1	LCK-3
.....................uggaucuuguucgacagcU..............................	155	1	JCK-1
.....................uggaucuuguuGgacagca..............................	9	1	LCK-3
.....................ugAaucuuguucgacagca..............................	1	1	JCK-2
.....................uggaucuuguucgCcagca..............................	3	1	LT-2
.....................uggaucuuguucgCcagca..............................	1	1	LCK-3
.....................uggaucuuguucgCcagca..............................	2	1	JCK-3
.....................uggaucuuguucgacaAca..............................	1	1	LCK-1
.....................uggaucuuguucgacagcG..............................	2	1	LT-3
.....................uggaucuuguucgacCgca..............................	1	1	LT-3
.....................uAgaucuuguucgacagca..............................	2	1	LCK-3
.....................uggCucuuguucgacagca..............................	1	1	LT-3
.....................uggaucuuguucgacagcU..............................	62	1	LCK-2
.....................uggaucuuguucgacagcU..............................	75	1	LT-1
.....................uggaucuuguucgacagcU..............................	211	1	LT-2
.....................uggaucuuguucgacagUa..............................	6	1	JCK-2
.....................uggGucuuguucgacagca..............................	1	1	JT-1
.....................uggaucuuguucgacagca..............................	1963	0	LCK-2
.....................uggaucuuguucgacagcU..............................	68	1	LCK-1
.....................uggaucuuguucgaUagca..............................	1	1	JCK-3
.....................uggaucuuguucgacagcC..............................	1	1	LCK-3
.....................uggaucuuguucgaUagca..............................	4	1	JCK-1
.....................uggaucuuguucgacagca..............................	1184	0	LT-2
.....................uNgaucuuguucgacagca..............................	1	1	LCK-1
.....................uggaucCuguucgacagca..............................	1	1	JT-1
.....................uggaCcuuguucgacagca..............................	2	1	JCK-3
.....................uggCucuuguucgacagca..............................	2	1	LCK-3
.....................uggGucuuguucgacagca..............................	1	1	JT-2
.....................uggaucuuguucgacaAca..............................	1	1	JT-1
.....................uggaucCuguucgacagca..............................	1	1	JCK-1
.....................uggaucuuguucgacagAa..............................	1	1	LT-3
.....................uggauUuuguucgacagca..............................	1	1	JCK-3
.....................uggaucuuguucgacagcU..............................	5	1	JT-3
.....................uggaucuuguucgaAagca..............................	1	1	LT-2
.....................uggaucuuguucgaUagca..............................	2	1	LCK-2
.....................uggaucuuguucgCcagca..............................	1	1	JT-1
.....................uggaucuuguucgacaAca..............................	1	1	LT-1
.....................uggaucuuguucgaUagca..............................	4	1	JCK-2
.....................ugAaucuuguucgacagca..............................	1	1	LT-2
.....................uggaucuuguucgacagcU..............................	162	1	JCK-3
.....................uAgaucuuguucgacagca..............................	1	1	JCK-2
.....................uggaucuuguuNgacagca..............................	5	1	JCK-1
.....................uggaucuuguucgacaUca..............................	3	1	JCK-2
.....................uggaucuuguucgacagcC..............................	1	1	JT-1
.....................uggaucuuguucgacagca..............................	529	0	JCK-1
.....................uggaucuuguucNacagca..............................	1	1	LCK-1
.....................uggaucuuguucgaNagca..............................	4	1	LCK-1
.....................uUgaucuuguucgacagca..............................	1	1	LT-3
.....................uggaucuuguuNgacagca..............................	2	1	JT-1
.....................uggCucuuguucgacagca..............................	1	1	JCK-3
.....................uNgaucuuguucgacagca..............................	2	1	JCK-3
.....................uggauUuuguucgacagca..............................	1	1	LCK-3
.....................Cggaucuuguucgacagca..............................	1	1	LCK-2
.....................uggaucCuguucgacagca..............................	1	1	LT-3
.....................uggCucuuguucgacagca..............................	1	1	JCK-2
.....................uggaucuuguucgacCgca..............................	1	1	LCK-3
.....................uggaucuuguucgNcagca..............................	6	1	LCK-1
.....................uggaucuuguucgacagGa..............................	1	1	JCK-2
.....................uggaucuuguucgacagcU..............................	56	1	JT-1
.....................uggaucuuguucgacagcU..............................	299	1	LCK-3
.....................uggaucuuguucgacagca..............................	2250	0	LCK-3
.....................uggaucuuguucgacaUca..............................	15	1	LCK-3
.....................uggaucuuguucgacagcC..............................	1	1	LCK-2
.....................uggaucuuguucgacagca..............................	606	0	JCK-3
.....................ugNaucuuguucgacagca..............................	5	1	LT-2
.....................uggaucuuguucgacagca..............................	237	0	JT-2
.....................uggaucuuguucgacagcU..............................	9	1	JT-2
.....................uggCucuuguucgacagcac.............................	1	1	LT-1

Mature Star

ggguguuguucauuuuacccucuggaucuuguucgacagcacucccaucaucuuuguucauguuugcgguuagggguguuauauauccacccccaaucucaaucucauaugugacaugcaccggaagugcugucgagcgugauuccgaaggugcuguucagcaacauacagauggaucgagcaacuacacugccugcuauauaugcccaucaucaucuucuacuuaaccuugcuuauuuauuuauuauuca			
......................uggaucuuguucgacGgcac	1	1	LT-1
......................uggaucuuguucgacagcUc	1	1	JCK-1
......................uggaucuuguucgCcagcac	6	1	LCK-3
......................uggaucuuguucgacCgcac	1	1	LT-2
......................uggaucuuguucgacagcaA	3	1	JCK-2
......................uggCucuuguucgacagcac	11	1	LT-2
......................uggaucuuguucgacGgcac	1	1	JCK-2
......................uggaucuuguucgacGgcac	1	1	LT-2
......................uggaucuuguucgacagcNc	1	1	LCK-1
......................uggaucuuguucgacagcaU	11	1	LT-2
......................uggaucuuAuucgacagcac	1	1	LCK-1
......................uNgaucuuguucgacagcac	1	1	JCK-1
......................uggauNuuguucgacagcac	2	1	LCK-1
......................uggaucuuguucgacaAcac	1	1	JCK-1
......................uggaucuuguucgCcagcac	1	1	JCK-1
......................Cggaucuuguucgacagcac	2	1	LT-2
......................uggaucuuguucgacagcUc	2	1	JCK-2
......................uggCucuuguucgacagcac	2	1	LT-3
......................uggaucuuguucgacagcCc	3	1	LT-2
......................uggaucuuguucgacagcUc	3	1	LT-3
......................uggaucuuguucgacagcac	796	0	LCK-2
......................uggaucuuguucgGcagcac	1	1	JCK-1
......................uggaucuuguucgaAagcac	1	1	LT-3
......................uggaucuugCucgacagcac	1	1	LT-2
......................uggaucuuguucgacagcac	72	0	JT-2
......................ugAaucuuguucgacagcac	1	1	LCK-3
......................uggaucuuguucgacagcac	508	0	JCK-3
......................uggCucuuguucgacagcac	2	1	JCK-2
......................uggCucuuguucgacagcac	5	1	LCK-3
......................uggaucuuguucgacagcaA	2	1	LT-3
......................uggaucuuguucgaAagcac	2	1	LCK-2
......................uggaucuuguucgacagcac	1028	0	LT-2
......................ugNaucuuguucgacagcac	4	1	LT-2
......................uggaucuuguucgacagcac	83	0	JT-3
......................uAgaucuuguucgacagcac	1	1	LT-3
......................uggaucuuguucAacagcac	1	1	LT-2
......................uggaucuuguucgacagcaU	16	1	LT-3
......................uggaucuuguucgacagcGc	1	1	LT-2
......................uggCucuuguucgacagcac	2	1	LCK-1
......................uggaucuuguucgacagcac	762	0	LT-3
......................uggaucuuguucgGcagcac	1	1	LCK-1
......................uggaucuuguucgacagcUc	1	1	LT-1
......................uggaucuuguucgaUagcac	1	1	JCK-1
......................uggaucuuguucgacagcaA	9	1	JCK-3
......................ugUaucuuguucgacagcac	1	1	JCK-3
......................uggaucuuguucgaNagcac	4	1	LCK-1
......................uggaucuuguucgaUagcac	1	1	LT-2
......................ugAaucuuguucgacagcac	1	1	LT-2
......................uggCucuuguucgacagcac	1	1	JT-3
......................uggaucuuguucgacagcCc	1	1	LCK-2
......................uggaucuuguucgacagcac	821	0	LCK-1
......................uggaucuuguucgacagcaU	12	1	JCK-3
......................uggaucuuguucgCcagcac	1	1	LT-3
......................uggaucuuguucgCcagcac	1	1	LCK-2
......................uggaucuuguucgacagcac	2109	0	LCK-3
......................uggaucuuguucgNcagcac	4	1	LCK-1
......................uggaucuuguucgaAagcac	1	1	JCK-1
......................uggaucuuguucgacagcac	564	0	LT-1
......................uggaucuuguucgacagcaU	1	1	LCK-3
......................uggaucuuguucgacagcUc	3	1	LT-2
......................uggaucuuguuNgacagcac	1	1	JT-2
......................ugUaucuuguucgacagcac	1	1	LT-3
......................uggaucuuguucgCcagcac	5	1	LT-2
......................uggaucuuguucgacagcaU	10	1	LT-1
......................uggaucuuguucgacagcac	727	0	JCK-2
......................uggaucuuguucgacagcaU	5	1	JCK-1
......................uggaucuuguucgacagcac	164	0	JT-1
......................uggaCcuuguucgacagcac	1	1	LCK-3
......................uggaucuuguucgacagcac	454	0	JCK-1
......................ugUaucuuguucgacagcac	1	1	LT-2

Mature Star

```
gggguguuguucauuuuacccucuggaucuuguucgacagcacucccaucaucuuuguucauguuugcgguuaggguguuauauauccacccccaaucucaaucucauaugugacaugcaccggaagugcugucgagcgugauuccgaaggugcuguucagcaacauacagauggaucgagcaacuacacugccugcuauauaugcccaucaucaucuucuacuuaaccuugcuuauuuauuuauuauuca
......................uggaucuuguucgaUagcac.............   3      1   JCK-3
......................uggaucuuguucgacagcaU.............   19     1   JCK-2
......................uggaucuuguucUacagcac.............   1      1   LT-1
......................uggaucuuguucgacagcCc.............   1      1   LCK-3
......................uAgaucuuguucgacagcac.............   1      1   LCK-3
......................uggaucuuguucgacagcCc.............   1      1   JT-3
......................uggCucuuguucgacagcac.............   4      1   LCK-2
......................ugAaucuuguucgacagcac.............   2      1   JCK-1
......................uggaucuuguucgaGagcac.............   1      1   LCK-1
......................uggaucuuguucgacagcNc.............   1      1   JCK-1
......................uggaucuuguucgacCgcac.............   2      1   JCK-1
......................uggaucuuguucgacagcCc.............   1      1   LT-1
......................uggaucuuguucgacagcUc.............   2      1   JCK-3
......................uggaucuuguucgacagcaA.............   1      1   JCK-1
......................uggaucuuguucgaUagcac.............   3      1   JCK-2
......................uggaucuuguucgacagUac.............   1      1   JCK-3
......................uggaucuuguucgaGagcacu............   1      1   JCK-3
......................uggNucuuguucgacagcacu............   1      1   JCK-2
......................uggaucuuguucgacCgcacu............   3      1   LT-3
......................uggaucuuguucgacaAcacu............   1      1   LCK-3
......................uggaucuuguucgacagUacu............   23     1   JCK-2
......................uggaucuuguucAacagcacu............   1      1   LCK-1
......................uggaucuuguucgacaUcacu............   2      1   JCK-1
......................uggaucCuguucgacagcacu............   5      1   JCK-3
......................uggaucuuguucgacCgcacu............   7      1   JCK-1
......................uggaucuuguucgGcagcacu............   2      1   JCK-1
......................uggaucuCguucgacagcacu............   2      1   JCK-3
......................uggaucuuguCcgacagcacu............   1      1   JCK-3
......................uggauNuuguucgacagcacu............   1      1   JCK-1
......................uggaucuuguucgacagcaUu............   17     1   LT-3
......................uggaucuugCucgacagcacu............   9      1   JCK-2
......................uggaucuuguucgacagcacA............   16     1   LT-1
......................uAgaucuuguucgacagcacu............   2      1   LCK-2
......................ugNaucuuguucgacagcacu............   1      1   LT-1
......................uggaucCuguucgacagcacu............   1      1   JT-1
......................uggaucuuguucgacagcacC............   1      1   LT-1
......................uggCucuuguucgacagcacu............   67     1   JCK-1
......................uggaucuCguucgacagcacu............   1      1   JCK-1
......................uggaucuuguucgacagcacu............   26265  0   JCK-3
......................Cggaucuuguucgacagcacu............   2      1   JCK-1
......................uggaucuuguucgacagcaUu............   38     1   LCK-3
......................uggaucuCguucgacagcacu............   1      1   LT-1
......................uggaucuuguucUacagcacu............   1      1   JCK-1
......................uggaucAuguucgacagcacu............   1      1   JCK-2
......................uggaucuGguucgacagcacu............   6      1   JCK-2
......................Aggaucuuguucgacagcacu............   4      1   LT-2
......................ugUaucuuguucgacagcacu............   3      1   JCK-2
......................uggaucuuguucAacagcacu............   6      1   JCK-1
......................uUgaucuuguucgacagcacu............   2      1   LCK-3
......................uggaucuuguucgacagcaUu............   38     1   JCK-2
......................uCgaucuuguucgacagcacu............   3      1   JCK-2
......................uggaucuuguucAacagcacu............   1      1   LCK-2
......................uggaucuuguuNgacagcacu............   121    1   JCK-1
......................uggaucuuguucgaUagcacu............   11     1   JCK-3
......................uggaucuuguucgacGgcacu............   1      1   LT-1
......................uNgaucuuguucgacagcacu............   31     1   JCK-1
......................uggaucuugCucgacagcacu............   3      1   JCK-1
......................uggaucuuguucgacagcaAu............   1      1   LCK-3
......................uggaucuuAuucgacagcacu............   1      1   JCK-3
......................uggaucuuguuNgacagcacu............   1      1   JCK-2
......................uggCucuuguucgacagcacu............   2      1   LCK-2
......................uNgaucuuguucgacagcacu............   2      1   LT-3
......................ugUaucuuguucgacagcacu............   7      1   JCK-3
......................uggaucuuguucgacagcacu............   35956  0   JCK-2
......................uNgaucuuguucgacagcacu............   37     1   JCK-2
......................Cggaucuuguucgacagcacu............   2      1   LT-3
......................ugUaucuuguucgacagcacu............   1      1   LCK-3
......................ugAaucuuguucgacagcacu............   2      1   LCK-3
......................uggaucuuguucgaAagcacu............   1      1   JCK-1
......................uggaucuuguucgacagcacA............   51     1   JCK-1
```

```
                      Mature                                                                                                       Star
ggguguuguucauuuuacccucuggaucuuguucgacagcacucccaucaucuuuguucauguuugcgguuaggguguuauauauccacccccaaucucaaucucauaugugacaugcaccggaagugcugucgagcgugauuccgaaggugcuguucagcaacauacagauggaucgagcaacuacacugccugcuauauaugcccaucaucaucuucuacuuaaccuugcuuauuuauuuauuauuca
......................uggaucuuguucgaUagcacu...............................................................................................................................................................................   12    1   JCK-1
......................uggaucuuguucgacGgcacu...............................................................................................................................................................................   5     1   JCK-2
......................uggaucuuguucgacagcacA...............................................................................................................................................................................   59    1   JCK-3
......................uggaucuuguuGgacagcacu...............................................................................................................................................................................   10    1   JCK-1
......................uggaCcuuguucgacagcacu...............................................................................................................................................................................   5     1   JCK-2
......................uggaucuuguucgacagcGcu...............................................................................................................................................................................   1     1   LT-3
......................uggaucuuguucgacagNacu...............................................................................................................................................................................   3     1   JCK-3
......................uggaucuuguucgacagcacA...............................................................................................................................................................................   61    1   LCK-3
......................uggaucuuguucgacagcaAu...............................................................................................................................................................................   2     1   JCK-3
......................uggCucuuguucgacagcacu...............................................................................................................................................................................   19    1   LT-3
......................uggaucuuguucgacagcacC...............................................................................................................................................................................   8     1   LT-2
......................uggaucuuguucgacagcaUu...............................................................................................................................................................................   18    1   LT-1
......................uggaucuuguucgacagcCcu...............................................................................................................................................................................   1     1   LCK-1
......................uggCucuuguucgacagcacu...............................................................................................................................................................................   18    1   LCK-3
......................uggaucuuUuucgacagcacu...............................................................................................................................................................................   3     1   JCK-2
......................uggaucuuguucgacagcGcu...............................................................................................................................................................................   2     1   JCK-3
......................uggaucuuAuucgacagcacu...............................................................................................................................................................................   2     1   JCK-1
......................uggaucuuguucAacagcacu...............................................................................................................................................................................   7     1   JCK-2
......................uggaucuuguucgacGgcacu...............................................................................................................................................................................   1     1   LCK-2
......................ugCaucuuguucgacagcacu...............................................................................................................................................................................   1     1   JCK-1
......................uggaucuuguucgacagcacu...............................................................................................................................................................................   423   0   JT-3
......................uggaucuuguucgacaUcacu...............................................................................................................................................................................   1     1   LT-2
......................uggaucuuguuGgacagcacu...............................................................................................................................................................................   1     1   JCK-3
......................uggaucCuguucgacagcacu...............................................................................................................................................................................   2     1   LT-2
......................uggaucuuguuGgacagcacu...............................................................................................................................................................................   1     1   JCK-2
......................Gggaucuuguucgacagcacu...............................................................................................................................................................................   1     1   JCK-3
......................uggaucuugCucgacagcacu...............................................................................................................................................................................   3     1   JCK-3
......................uAgaucuuguucgacagcacu...............................................................................................................................................................................   3     1   LT-3
......................uggaucuuguucgacagcacG...............................................................................................................................................................................   12    1   JCK-3
......................uggaucuuguucgacagcCcu...............................................................................................................................................................................   2     1   LCK-3
......................uggaCcuuguucgacagcacu...............................................................................................................................................................................   1     1   LCK-2
......................uggaucuuguucgacagcUcu...............................................................................................................................................................................   2     1   LT-1
......................uggaucuuguucgacagcacA...............................................................................................................................................................................   37    1   JT-1
......................uggaucuuguucgacagcacA...............................................................................................................................................................................   79    1   JCK-2
......................uggaucuuguuAgacagcacu...............................................................................................................................................................................   1     1   JCK-2
......................uggCucuuguucgacagcacu...............................................................................................................................................................................   75    1   JCK-3
......................uggaucuuAuucgacagcacu...............................................................................................................................................................................   10    1   JCK-2
......................uggaucuuguucCacagcacu...............................................................................................................................................................................   1     1   JCK-2
......................uggaCcuuguucgacagcacu...............................................................................................................................................................................   4     1   JCK-3
......................uggCucuuguucgacagcacu...............................................................................................................................................................................   4     1   JT-1
......................uggaucCuguucgacagcacu...............................................................................................................................................................................   2     1   LCK-3
......................uggaucuuguucgacCgcacu...............................................................................................................................................................................   13    1   JCK-2
......................uggaucuuguucgacagcacu...............................................................................................................................................................................   4453  0   LT-3
......................uggaucuCguucgacagcacu...............................................................................................................................................................................   1     1   LCK-2
......................uggaucCuguucgacagcacu...............................................................................................................................................................................   7     1   JCK-2
......................uggaucuCguucgacagcacu...............................................................................................................................................................................   7     1   JCK-2
......................Cggaucuuguucgacagcacu...............................................................................................................................................................................   2     1   LT-2
......................uggaucuuguucgCcagcacu...............................................................................................................................................................................   10    1   LT-3
......................uCgaucuuguucgacagcacu...............................................................................................................................................................................   1     1   LT-2
......................ugAaucuuguucgacagcacu...............................................................................................................................................................................   2     1   LT-1
......................uggCucuuguucgacagcacu...............................................................................................................................................................................   55    1   LT-2
......................uggaucuuguCcgacagcacu...............................................................................................................................................................................   2     1   JCK-1
......................uggaucuuguucgacGgcacu...............................................................................................................................................................................   2     1   JCK-1
......................uggauNuuguucgacagcacu...............................................................................................................................................................................   1     1   LCK-1
......................ugAaucuuguucgacagcacu...............................................................................................................................................................................   11    1   JCK-3
......................Aggaucuuguucgacagcacu...............................................................................................................................................................................   1     1   JCK-2
......................uggaucuuguucgacagcacA...............................................................................................................................................................................   24    1   LT-3
......................uNgaucuuguucgacagcacu...............................................................................................................................................................................   29    1   JCK-3
......................uggaucuuguucgacCgcacu...............................................................................................................................................................................   1     1   LCK-2
......................uggaucuuguucgacagcaNu...............................................................................................................................................................................   23    1   JCK-1
......................uggaucuuguucgacagcCcu...............................................................................................................................................................................   24    1   JCK-2
......................uggaucuuguucgacCgcacu...............................................................................................................................................................................   10    1   JCK-3
......................Cggaucuuguucgacagcacu...............................................................................................................................................................................   8     1   JCK-2
......................uggaucuuguucgacagUacu...............................................................................................................................................................................   19    1   JCK-3
......................uggaucuuguucgacagcaAu...............................................................................................................................................................................   12    1   JCK-1
......................uggaucuuguucgacagcCcu...............................................................................................................................................................................   1     1   JT-1
......................uggaucuuguucgacagcacC...............................................................................................................................................................................   10    1   JCK-3
......................uggaucuuguucAacagcacu...............................................................................................................................................................................   1     1   JCK-3
......................uggaucuuAuucgacagcacu...............................................................................................................................................................................   1     1   LT-2
......................uggaucuuguucgaNagcacu...............................................................................................................................................................................   4     1   LCK-1
```

Mature / **Star**

gggguguuguucauuuuacccucuggaucuuguucgacagcacucccaucaucuuuguucauguuugcgguuagggguguuauauauccacccccaaucucaaucucauaugugacaugcaccggaagugcugucgagcgugauuccgaaggugcuguucagcaacauacagauggaucgagcaacuacacugccugcuauauaugcccaucaucaucuucuacuuaaccuugcuuauuuauuuauuauuca

uggaucuuguucgacagcacu	1264	0	LCK-1
uggaucuuguucgacagcacC	3	1	LT-3
uggaucuuguucgacagcaUu	13	1	JCK-1
uggaucuuguucgacagcacG	2	1	LT-3
uggaucuuguucgCcagcacu	8	1	LT-1
uggaucuuguucgacagcCcu	2	1	LT-3
uAgaucuuguucgacagcacu	3	1	LT-2
uggaucGuguucgacagcacu	1	1	JCK-3
uUgaucuuguucgacagcacu	6	1	JCK-3
uggGucuuguucgacagcacu	4	1	JCK-3
uggNucuuguucgacagcacu	1	1	LCK-1
uggaucuuguucgCcagcacu	2	1	LCK-2
ugAaucuuguucgacagcacu	2	1	LT-2
uggaucuuguucgacagcacu	1314	0	LCK-2
uggaucCuguucgacagcacu	1	1	JT-3
ugAaucuuguucgacagcacu	6	1	JCK-1
uggaucuuguucgacagcacu	277	0	JT-2
uggaucuuguucgacaAcacu	2	1	JCK-3
uggaucuuguucgacagcaAu	3	1	LT-1
uggaucuuguucgacagcCcu	17	1	JCK-3
uUgaucuuguucgacagcacu	1	1	LT-2
uggaucuuguucgacagcaAu	1	1	LCK-2
uggaucuuguucgacaAcacu	1	1	LT-1
uggaucuuguuNgacagcacu	1	1	JT-1
uggauUuuguucgacagcacu	4	1	JCK-2
uCgaucuuguucgacagcacu	1	1	JCK-3
uggCucuuguucgacagcacu	10	1	LT-1
uggaucuuguucgacagcaAu	3	1	JCK-2
uggaucuuguucgacagcacu	20810	0	JCK-1
uggaucuuguucgacagcNcu	10	1	JCK-1
uggauUuuguucgacagcacu	1	1	JCK-1
uAgaucuuguucgacagcacu	1	1	LT-1
uUgaucuuguucgacagcacu	1	1	JCK-1
uggaucuuguucgacaCcacu	1	1	JCK-1
uggaucuuguucgCcagcacu	47	1	JCK-1
uggaucuuguucgacagcGcu	1	1	JCK-1
uAgaucuuguucgacagcacu	10	1	JCK-2
uggaucuuguucgacGgcacu	10	1	JCK-3
uggaucuuguucgacagcacu	5544	0	LCK-3
ugAaucuuguucgacagcacu	8	1	JCK-2
uggaucuuguucgacagcCcu	1	1	LCK-2
uggaucuuguucgacCgcacu	3	1	LT-2
uggaucCuguucgacagcacu	2	1	JCK-1
uggaucuuguucgacagcaGu	1	1	JCK-1
uggaucuuguucgCcagcacu	48	1	JCK-3
Cggaucuuguucgacagcacu	2	1	JCK-3
uggaucuuAuucgacagcacu	1	1	LT-3
uggaucuuguucUacagcacu	4	1	JCK-2
uggaucuuguucCacagcacu	4	1	LCK-3
uggaucuuUuucgacagcacu	3	1	JCK-3
uggCucuuguucgacagcacu	1	1	JT-2
uggaucuuguucgacagcacC	4	1	JT-3
uggGucuuguucgacagcacu	1	1	LCK-3
uggaucuuguucgacagcacC	22	1	JCK-2
uggaucuuguucgacagcacG	6	1	LCK-3
uAgaucuuguucgacagcacu	7	1	JCK-3
uggaucuugCucgacagcacu	6	1	LCK-3
uUgaucuuguucgacagcacu	3	1	JCK-2
ugUaucuuguucgacagcacu	2	1	JCK-1
uggaucuuguucgacagcCcu	7	1	LT-2
ugNaucuuguucgacagcacu	9	1	LT-2
uggaucuuNuucgacagcacu	2	1	JCK-3
uggaucuuguuAgacagcacu	1	1	JCK-1
uggaucuuguucgacagcacu	4196	0	LT-2
uggaucuuguucgacGgcacu	1	1	LT-2
uggaucuuguucgacaUcacu	5	1	JCK-2
uggaucuuguucgaAagcacu	5	1	JCK-2
uggaucuuguucgacagcCcu	3	1	LT-1
uggaucuuguucNacagcacu	1	1	LCK-1
uggaucuuguucgacagcacA	50	1	LT-2

Mature **Star**

ggguguuguucauuuuacccucuggaucuuguucgacagcacucccaucaucuuuguucauguuugcgguuagggguguuauauauccacccccaaucucaaucucauaugugacaugcaccggaagugcugucgagcgugauuccgaaggugcuguucagcaacauacagauggaucgagcaacuacacugccugcuauauaugcccaucaucaucuucuacuuaaccuugcuuauuuauuuauuauuca

......................uggaucuuguucgacagcaUu	17	1	JCK-3
......................uggaucuuguucgNcagcacu	5	1	LCK-1
......................uggaCcuuguucgacagcacu	1	1	JCK-1
......................uggaucuuguucgGcagcacu	2	1	LCK-1
......................uggaucuuguucgacagcacC	2	1	LCK-3
......................uggGucuuguucgacagcacu	3	1	JCK-2
......................uggaucuuguucgacGgcacu	1	1	LCK-3
......................uggaucuuguGcgacagcacu	3	1	JCK-1
......................uggaucuuguucgacagcacN	6	1	JCK-1
......................uggaucuuguucUacagcacu	6	1	JCK-3
......................uAgaucuuguucgacagcacu	1	1	JT-1
......................ugUaucuuguucgacagcacu	1	1	LT-1
......................uggaucuuguucgacagcacu	2933	0	LT-1
......................uggaucuuguucgaUagcacu	2	1	LT-1
......................uggaucuuguucgacagcaUu	7	1	JT-1
......................uggNucuuguucgacagcacu	4	1	JCK-3
......................uggaucuuguucgacagcacG	15	1	JCK-2
......................uggaucuuguucgCcagcacu	6	1	LCK-3
......................uggaucuuguucgGcagcacu	2	1	JCK-3
......................uggaucuuguucgacaAcacu	9	1	JCK-2
......................ugAaucuuguucgacagcacu	1	1	LT-3
......................uggaucuuguuGgacagcacu	1	1	LT-3
......................uggaucuuguucgaUagcacu	2	1	LT-3
......................uggaucuuguucgacagcacG	12	1	JCK-1
......................uggaucuuguucgacagcCcu	11	1	JCK-1
......................uggaucuuguucgCcagcacu	80	1	JCK-2
......................Cggaucuuguucgacagcacu	1	1	LCK-3
......................uggaucuuguucgacagcacG	2	1	LT-1
......................uggaCcuuguucgacagcacu	1	1	LCK-3
......................uggaucuuguucgacagcacC	2	1	JT-2
......................uAgaucuuguucgacagcacu	9	1	JCK-1
......................uggaucuuguucgacagcaAu	2	1	LT-3
......................uggaucuuguucgacaUcacu	4	1	JCK-3
......................uggaucuuguucgacagcaAu	3	1	LT-2
......................uggaucuuguucgacagcacC	5	1	JCK-1
......................uggaucuuguucgacagcUcu	1	1	JCK-3
......................uggaucuuguucgacaAcacu	8	1	JCK-1
......................uggCucuuguucgacagcacu	104	1	JCK-2
......................ugNaucuuguucgacagcacu	2	1	LCK-1
......................uggaucuuguucgCcagcacu	2	1	JT-3
......................uggaucuuAuucgacagcacu	3	1	LCK-3
......................uggaucuuguucgaUagcacu	26	1	JCK-2
......................uggaucuuguuAgacagcacu	1	1	LCK-3
......................uggaucuuguucgacagUacu	16	1	JCK-1
......................uggaucuuguucgacagcUcu	1	1	JCK-2
......................uggaucuuguucgacagUacu	1	1	LT-1
......................uggaucuuguucgaAagcacu	9	1	JCK-3
......................uggNucuuguucgacagcacu	21	1	JCK-1
......................uggGucuuguucgacagcacu	9	1	JCK-1
......................uggaucuuguucgacagcacu	1125	0	JT-1
......................uggaucuuguucgacagAacu	1	1	JCK-2
......................uggaucuuguCcgacagcacu	1	1	LT-3
......................uggaucuuguucgacagcGcu	7	1	JCK-2
......................uggaucuuguucgCcagcacu	2	1	JT-1
......................uggaucuuguucgacagGacu	7	1	JCK-1
......................uggaucuuguCcgacagcacu	2	1	JCK-2
......................uggaucuuguucgacagcaUu	17	1	LT-2
......................uggaucuuCuucgacagcacu	1	1	JCK-3
......................uggCucuuguucgacagcacu	1	1	LCK-1
......................uAgaucuuguucgacagcacu	7	1	LCK-3
......................uggauUuuguucgacagcacu	4	1	JCK-3
......................uggaucuuguucgCcagcacu	16	1	LT-2
......................uggaucuuguucgacaAcacuc	1	1	JCK-1
......................uggaucuuguucgacagcacuA	12	1	LT-2
......................uggaucuuguucgacagcaNuc	5	1	JCK-1
......................uggaucuuguucgacagcaUuc	10	1	JCK-3
......................uggaucuuguucgacagcacuc	8479	0	JCK-1
......................uggaucuuguucgacagGacuc	2	1	JCK-2
......................uggaucuuguucgacagcacuc	1033	0	LT-2
......................uggaucuuguucgacagUacuc	5	1	JCK-2

```
                      Mature                                                                                                       Star
ggguguuguucauuuuacccucuggaucuuguucgacagcacucccaucaucuuuguucauguuugcgguuaggguguuauauauccacccccaaucucaaucucauaugugacaugcaccggaagugcugucgagcgugauuccgaaggugcuguucagcaacauacagauggaucgagcaacuacacugccugcuauauaugcccaucaucaucuucuacuuaaccuugcuuauuuauuuauuauuca
......................uggaucuuguucUacagcacuc..............................................................................................................................................................................................................  2      1    JCK-1
......................uggaucuCguucgacagcacuc..............................................................................................................................................................................................................  1      1    LT-1
......................uggaucuuguucgacaUcacuc..............................................................................................................................................................................................................  4      1    JCK-2
......................uggCucuuguucgacagcacuc..............................................................................................................................................................................................................  43     1    JCK-2
......................uggaucuuguucgacagcCcuc..............................................................................................................................................................................................................  9      1    JCK-2
......................uggaucuuguucgacagcCcuc..............................................................................................................................................................................................................  3      1    JCK-1
......................uggaucuuAuucgacagcacuc..............................................................................................................................................................................................................  1      1    JCK-1
......................uggaucuuguucgacagcacuA..............................................................................................................................................................................................................  36     1    LCK-3
......................uggaucuuguucgaUagcacuc..............................................................................................................................................................................................................  26     1    JCK-3
......................uggaucuuUuucgacagcacuc..............................................................................................................................................................................................................  3      1    JCK-2
......................uggauUuuguucgacagcacuc..............................................................................................................................................................................................................  4      1    JCK-2
......................uggaucuuguucUacagcacuc..............................................................................................................................................................................................................  1      1    JCK-2
......................uggaucuuguuGgacagcacuc..............................................................................................................................................................................................................  3      1    JCK-1
......................uggaucuuguucgacagcacuG..............................................................................................................................................................................................................  12     1    JCK-2
......................uggGucuuguucgacagcacuc..............................................................................................................................................................................................................  2      1    JCK-2
......................uggaucuuguucgacagcNcuc..............................................................................................................................................................................................................  3      1    JCK-1
......................uggaCcuuguucgacagcacuc..............................................................................................................................................................................................................  1      1    LT-1
......................uggaucCuguucgacagcacuc..............................................................................................................................................................................................................  1      1    LT-3
......................uggaucuuguucgacaAcacuc..............................................................................................................................................................................................................  4      1    JCK-2
......................uggaucuuguucgacagcacuc..............................................................................................................................................................................................................  14605  0    JCK-2
......................uggaCcuuguucgacagcacuc..............................................................................................................................................................................................................  1      1    JCK-1
......................uggaucuuUuucgacagcacuc..............................................................................................................................................................................................................  3      1    JCK-3
......................uggaucuuguGcgacagcacuc..............................................................................................................................................................................................................  5      1    JCK-1
......................uggaucuuguucgaAagcacuc..............................................................................................................................................................................................................  3      1    JCK-3
......................Cggaucuuguucgacagcacuc..............................................................................................................................................................................................................  2      1    JCK-1
......................uggaucuCguucgacagcacuc..............................................................................................................................................................................................................  1      1    JCK-1
......................ugAaucuuguucgacagcacuc..............................................................................................................................................................................................................  5      1    JCK-3
......................uggaucuuNuucgacagcacuc..............................................................................................................................................................................................................  1      1    JCK-3
......................uggaCcuuguucgacagcacuc..............................................................................................................................................................................................................  3      1    JCK-3
......................uggNucuuguucgacagcacuc..............................................................................................................................................................................................................  1      1    JCK-3
......................uAgaucuuguucgacagcacuc..............................................................................................................................................................................................................  2      1    LT-2
......................uggaucuuguucgacagcaUuc..............................................................................................................................................................................................................  7      1    JCK-1
......................uggaucuuguucgacagcCcuc..............................................................................................................................................................................................................  1      1    LT-2
......................uUgaucuuguucgacagcacuc..............................................................................................................................................................................................................  1      1    LT-1
......................uggaucuuguucgacagcacuc..............................................................................................................................................................................................................  188    0    JT-1
......................uggaucuugCucgacagcacuc..............................................................................................................................................................................................................  2      1    LT-1
......................ugNaucuuguucgacagcacuc..............................................................................................................................................................................................................  2      1    LT-2
......................ugAaucuuguucgacagcacuc..............................................................................................................................................................................................................  4      1    JCK-2
......................uggaucuuguucgacagcacuA..............................................................................................................................................................................................................  14     1    LCK-1
......................uUgaucuuguucgacagcacuc..............................................................................................................................................................................................................  1      1    JCK-2
......................uggaucuuguucgaUagcacuc..............................................................................................................................................................................................................  42     1    JCK-2
......................uAgaucuuguucgacagcacuc..............................................................................................................................................................................................................  3      1    JCK-1
......................uggaucuuAuucgacagcacuc..............................................................................................................................................................................................................  1      1    JCK-3
......................uggaucuuguucgacagcacuU..............................................................................................................................................................................................................  1      1    LCK-1
......................uggaucuuguucgacagcacuc..............................................................................................................................................................................................................  2355   0    LT-3
......................uggaucuuguucCacagcacuc..............................................................................................................................................................................................................  5      1    JCK-2
......................uggGucuuguucgacagcacuc..............................................................................................................................................................................................................  4      1    JCK-1
......................uggaucuuguucgacagcacuA..............................................................................................................................................................................................................  47     1    JCK-1
......................uggaucuuguucgacGgcacuc..............................................................................................................................................................................................................  1      1    LT-1
......................uAgaucuuguucgacagcacuc..............................................................................................................................................................................................................  3      1    JCK-3
......................uggaucuuguucCacagcacuc..............................................................................................................................................................................................................  1      1    JCK-3
......................uggaucuuguucgacagcacuU..............................................................................................................................................................................................................  120    1    JCK-2
......................uggaucuuguucgacagcacuU..............................................................................................................................................................................................................  25     1    LT-2
......................uggCucuuguucgacagcacuc..............................................................................................................................................................................................................  8      1    LT-2
......................uggaucuuguucAacagcacuc..............................................................................................................................................................................................................  2      1    JCK-3
......................uggaucuuUuucgacagcacuc..............................................................................................................................................................................................................  1      1    JCK-1
......................uggaucuuguucgacGgcacuc..............................................................................................................................................................................................................  2      1    JCK-2
......................uggaucuuguucgacaAcacuc..............................................................................................................................................................................................................  1      1    LT-1
......................uggaucuuguucgacagcacuA..............................................................................................................................................................................................................  19     1    LT-1
......................uggaucuuguucgacagcacuN..............................................................................................................................................................................................................  4      1    JCK-1
......................uggaucuuguucgacaCcacuc..............................................................................................................................................................................................................  1      1    LT-1
......................uggaucuuguucgacGgcacuc..............................................................................................................................................................................................................  2      1    LT-2
......................uUgaucuuguucgacagcacuc..............................................................................................................................................................................................................  1      1    LCK-3
......................uggaucuuguCcgacagcacuc..............................................................................................................................................................................................................  1      1    JCK-3
......................uAgaucuuguucgacagcacuc..............................................................................................................................................................................................................  1      1    LCK-3
......................uggaucuuguucgacCgcacuc..............................................................................................................................................................................................................  5      1    JCK-3
......................uggaCcuuguucgacagcacuc..............................................................................................................................................................................................................  2      1    JCK-2
......................ugAaucuuguucgacagcacuc..............................................................................................................................................................................................................  1      1    LT-1
......................uggaucuuguucgacaUcacuc..............................................................................................................................................................................................................  1      1    JCK-1
......................uggaucuuguucgacagUacuc..............................................................................................................................................................................................................  4      1    JCK-1
```

```
                      Mature                                                                                                         Star
ggguguuguucauuuuacccucuggaucuuguucgacagcacucccaucaucuuuguucauguuugcgguuaggguguuauauauccacccccaaucucaaucucauaugugacaugcaccggaagugcugucgagcgugauuccgaaggugcuguucagcaacauacagauggaucgagcaacuacacugccugcuauauaugcccaucaucaucuucuacuuaaccuugcuuauuuauuuauuauuca
......................uggaucuuguucgCcagcacuc..............................................................................................................................................................................................................    2       1   LT-1
......................uggCucuuguucgacagcacuc..............................................................................................................................................................................................................    19      1   JCK-1
......................uggaucCuguucgacagcacuc..............................................................................................................................................................................................................    2       1   LCK-3
......................uggaucuuAuucgacagcacuc..............................................................................................................................................................................................................    1       1   LT-3
......................uggaucuuguucgacaAcacuc..............................................................................................................................................................................................................    1       1   LT-3
......................uggaucuuguucgacagcacuA..............................................................................................................................................................................................................    61      1   JCK-2
......................uggCucuuguucgacagcacuc..............................................................................................................................................................................................................    2       1   JT-1
......................uNgaucuuguucgacagcacuc..............................................................................................................................................................................................................    1       1   LT-3
......................uggCucuuguucgacagcacuc..............................................................................................................................................................................................................    7       1   LT-3
......................uggaucuuguucAacagcacuc..............................................................................................................................................................................................................    1       1   LT-1
......................uUgaucuuguucgacagcacuc..............................................................................................................................................................................................................    1       1   JCK-1
......................uAgaucuuguucgacagcacuc..............................................................................................................................................................................................................    4       1   JCK-2
......................uggaucuuguucgacagcacuU..............................................................................................................................................................................................................    63      1   JCK-1
......................uggaucCuguucgacagcacuc..............................................................................................................................................................................................................    1       1   JCK-2
......................uggaucuuguucgUcagcacuc..............................................................................................................................................................................................................    1       1   JCK-3
......................uggaucuuguucgUcagcacuc..............................................................................................................................................................................................................    1       1   JCK-1
......................uggaucuugCucgacagcacuc..............................................................................................................................................................................................................    5       1   JCK-2
......................uggaucuuguucUacagcacuc..............................................................................................................................................................................................................    2       1   LT-1
......................uggaucuuguucCacagcacuc..............................................................................................................................................................................................................    2       1   JCK-1
......................uggauUuuguucgacagcacuc..............................................................................................................................................................................................................    2       1   JCK-3
......................uNgaucuuguucgacagcacuc..............................................................................................................................................................................................................    11      1   JCK-1
......................uggNucuuguucgacagcacuc..............................................................................................................................................................................................................    8       1   JCK-1
......................uggaucuuguucgCcagcacuc..............................................................................................................................................................................................................    1       1   LT-2
......................uggaucuuguucgUcagcacuc..............................................................................................................................................................................................................    3       1   JCK-2
......................uggaucuuguucgCcagcacuc..............................................................................................................................................................................................................    5       1   LT-3
......................uggaucuuguucgacagcacuG..............................................................................................................................................................................................................    9       1   JCK-1
......................uggaucuuguucgacagUacuc..............................................................................................................................................................................................................    3       1   JCK-3
......................uggaucuuguucgacagcacuc..............................................................................................................................................................................................................    58      0   JT-2
......................uggaucuuguucgacagAacuc..............................................................................................................................................................................................................    1       1   JCK-1
......................uggaucuuguucgacagcGcuc..............................................................................................................................................................................................................    1       1   JCK-3
......................uggaucuuguucgacagcacuU..............................................................................................................................................................................................................    27      1   LT-1
......................uggaucuuguucgaUagcacuc..............................................................................................................................................................................................................    2       1   LT-1
......................uNgaucuuguucgacagcacuc..............................................................................................................................................................................................................    9       1   JCK-3
......................uggaucuCguucgacagcacuc..............................................................................................................................................................................................................    1       1   JCK-3
......................Cggaucuuguucgacagcacuc..............................................................................................................................................................................................................    1       1   LT-2
......................uggaucuuguucgacagNacuc..............................................................................................................................................................................................................    1       1   JCK-3
......................uggGucuuguucgacagcacuc..............................................................................................................................................................................................................    1       1   LT-3
......................uggauUuuguucgacagcacuc..............................................................................................................................................................................................................    3       1   JCK-1
......................uggaucuuguucgacagcacuU..............................................................................................................................................................................................................    31      1   LCK-3
......................uggaucuugCucgacagcacuc..............................................................................................................................................................................................................    5       1   JCK-1
......................uggaucuuguucgacagcacNc..............................................................................................................................................................................................................    4       1   JCK-1
......................Cggaucuuguucgacagcacuc..............................................................................................................................................................................................................    3       1   JCK-3
......................uggaucuuguucgaAagcacuc..............................................................................................................................................................................................................    5       1   JCK-2
......................uggaucuuguucgGcagcacuc..............................................................................................................................................................................................................    1       1   LCK-1
......................uggaucuuguucgacagGacuc..............................................................................................................................................................................................................    6       1   JCK-1
......................uggaucuuguucgacagcacuc..............................................................................................................................................................................................................    955     0   LCK-3
......................uggaucuuguucgacCgcacuc..............................................................................................................................................................................................................    8       1   JCK-2
......................uUgaucuuguucgacagcacuc..............................................................................................................................................................................................................    2       1   JCK-3
......................uggaucuuguucgacagcCcuc..............................................................................................................................................................................................................    2       1   LT-1
......................uggaucuuguucgCcagcacuc..............................................................................................................................................................................................................    11      1   JCK-3
......................Cggaucuuguucgacagcacuc..............................................................................................................................................................................................................    3       1   JCK-2
......................uNgaucuuguucgacagcacuc..............................................................................................................................................................................................................    15      1   JCK-2
......................uggaucuuguucgacagcacuG..............................................................................................................................................................................................................    2       1   JCK-3
......................uggaucuuguucgacagcCcuc..............................................................................................................................................................................................................    1       1   JCK-3
......................uggaucuuguuNgacagcacuc..............................................................................................................................................................................................................    29      1   JCK-1
......................uggaucuCguucgacagcacuc..............................................................................................................................................................................................................    1       1   LCK-3
......................uggaucuuguucgacagcacuc..............................................................................................................................................................................................................    69      0   JT-3
......................uggaucuuguucgacagcGcuc..............................................................................................................................................................................................................    2       1   JCK-1
......................uggaucuuguucgacCgcacuc..............................................................................................................................................................................................................    3       1   JCK-1
......................uggaucuuguucgacagcacuc..............................................................................................................................................................................................................    84      0   LCK-2
......................uggaucuuAuucgacagcacuc..............................................................................................................................................................................................................    2       1   JCK-2
......................uggCucuuguucgacagcacuc..............................................................................................................................................................................................................    24      1   JCK-3
......................uggaucuuguucgacagcaUuc..............................................................................................................................................................................................................    14      1   JCK-2
......................uggaucuuguucgacCgcacuc..............................................................................................................................................................................................................    1       1   LCK-2
......................uggaucuuguucgCcagcacuc..............................................................................................................................................................................................................    29      1   JCK-2
......................uggaucuuguucgaUagcacuc..............................................................................................................................................................................................................    10      1   JCK-1
......................uggCucuuguucgacagcacuc..............................................................................................................................................................................................................    3       1   LT-1
......................uggaucuuguucgacagcacuc..............................................................................................................................................................................................................    10812   0   JCK-3
......................uggaucuuguucgacagcacuG..............................................................................................................................................................................................................    3       1   LT-1
......................uggaucuuguucgacagcacuU..............................................................................................................................................................................................................    81      1   JCK-3
```

Mature   Star

gggguguuguucauuuuacccucuggaucuuguucgacagcacucccaucaucuuuguucauguuugcgguuagggguguuauauauccacccccaaucucaaucucauaugugacaugcaccggaagugcugucgagcgugauuccgaaggugcuguucagcaacauacagauggaucgagcaacuacacugccugcuauauaugcccaucaucaucuucuacuuaaccuugcuuauuuauuuauuauuca

.......................uggaucuuguucgacaUcacuc...	1	1	JCK-3
.......................uggaucuuguucUacagcacuc...	2	1	JCK-3
.......................uggaucuuguucgacagcacuA...	24	1	LT-3
.......................uggaucuuguucgCcagcacuc...	17	1	JCK-1
.......................uggaucCuguucgacagcacuc...	3	1	JCK-3
.......................uggaucuuguucgacGgcacuc...	4	1	JCK-3
.......................uggaucuuguucgacagcacuA...	9	1	JT-1
.......................uggaucuuguucgacagcacuc...	74	0	LCK-1
.......................uggaucuuguucAacagcacuc...	1	1	LT-2
.......................uggaucuuguucgaUagcacuc...	1	1	LT-3
.......................uggaucuuguucgacGgcacuc...	1	1	JCK-1
.......................uggaucuuguucgacagcacuA...	11	1	LCK-2
.......................uggaucuuguucgacagcacuU...	35	1	LT-3
.......................uggaucuuguucAacagcacuc...	1	1	JCK-1
.......................uggaucuuguucgacCgcacuc...	1	1	LT-3
.......................uggaucuugCucgacagcacuc...	4	1	JCK-3
.......................uggaucuuguucgacagcacuG...	1	1	LT-3
.......................uggCucuuguucgacagcacuc...	1	1	LCK-3
.......................uggaucuuguucgacagcacCc...	2	1	JCK-3
.......................ugAaucuuguucgacagcacuc...	2	1	JCK-1
.......................uggaucuuguucgacagcacCc...	1	1	LT-3
.......................uAgaucuuguucgacagcacuc...	1	1	LT-3
.......................uggaucuuguucgacagcaAuc...	1	1	JCK-1
.......................uggaucuuguucgacagcacAc...	1	1	LT-1
.......................uggaucuuguucgacagcCcuc...	1	1	LT-3
.......................uggaucuuguucgacagcacGc...	1	1	JCK-1
.......................uggaucuuguucgacagcacuA...	67	1	JCK-3
.......................uggaucuuguucAacagcacuc...	1	1	JCK-2
.......................uggaucuuguucgacagcacAc...	1	1	JCK-2
.......................uAgaucuuguucgacagcacuc...	2	1	LT-1
.......................uggaucuuguucgacagcaUuc...	1	1	LT-1
.......................uNgaucuuguucgacagcacuc...	1	1	LCK-3
.......................uggaucuuguucgacagcacCc...	2	1	JCK-2
.......................ugAaucuuguucgacagcacuc...	5	1	LT-3
.......................uggaucuuguucgacagcacuc...	2023	0	LT-1
.......................uggaucuuguucgacagcacucA..	64	1	JCK-2
.......................uggaucuuguucgacagcacucA..	14	1	LT-1
.......................uggaucuuguucgacagcacucc..	5	0	LCK-3
.......................uggaucuuguucgacagcacuUc..	28	1	LCK-2
.......................uggaucuuguucgacagcacucc..	1	0	LT-1
.......................uggaucuuguucgacagGacucc..	1	1	JCK-1
.......................uggaucuuguucgacagcacuAc..	6	1	LT-2
.......................uggaucuuguucgacagcacucc..	4	0	JCK-2
.......................uggaucuuguucgacagcacucc..	1	0	LT-3
.......................uggaucuuguucgacagcacucG..	1	1	JCK-3
.......................uggaucuuguucgacagcacucA..	8	1	LT-2
.......................uggaucuuguucgacagcacucN..	1	1	JCK-3
.......................uggaucuuguucgacagcacuUc..	2	1	JCK-1
.......................uggaucuuguucgacagcacucA..	18	1	LCK-3
.......................uggaucuuguucgacagcacuUc..	23	1	LCK-1
.......................uggaucuuguucgacagcacucG..	4	1	JCK-2
.......................uggaucuuguucgacagcacucG..	1	1	LT-3
.......................uggaucuuguucgacagcacuGc..	1	1	JCK-2
.......................uggaucuuguucgacagcacucA..	59	1	JCK-1
.......................uggaucuuguucgacagcacuUc..	4	1	JCK-3
.......................uggaucuuguucgacagcacucG..	5	1	LT-2
.......................uggaucuuguucgacagcacucc..	3	0	JCK-1
.......................uggaucuuguucgacagcacucc..	3	0	JCK-3
.......................uggaucuuguucgacagcacuGc..	1	1	JCK-1
.......................uggaucuuguucgacagcacucA..	11	1	LT-3
.......................uggaucuuguucgacagcacucA..	52	1	JCK-3
.......................uggaucuuguucgacagcacuccc...	3	0	JCK-1
.......................uggaucuuguucgacagcacuccc...	3	0	JCK-2
.......................uggaucuuguucgacagcacuccU...	3	1	LT-1
.......................uggaucuuguucgacagcacuccc...	3	0	JCK-3
.......................uggaucuuguucgacagcacuccU...	4	1	LT-2
.......................uggaucuuguucgacagcacucUca..	1	1	LT-3
.......................uggaucuuguucgacagcacucccU..	1	1	LT-1
.......................uggaucuuguucgacagcacuccGa..	2	1	JCK-2
.......................uggaucuuguucgacagcacuccGa..	2	1	JCK-1

Mature — Star

ggguguuguucauuuuacccucuggaucuuguucgacagcacucccaucaucuuuguucauguuugcgguuaggguguuauauauccacccccaaucucaaucucauaugugacaugcaccggaagugcugucgagcgugauuccgaaggugcuguucagcaacauacagauggaucgagcaacuacacugccugcuauauaugcccaucaucaucuucuacuuaaccuugcuuauuuauuuauuauuca			
......................uggaucuuguucgacagcacucUcau..	1	1	LCK-3
.......................ggaucuuguucgacagca...	10	0	JT-1
.......................ggaucuuguucgacagca...	2	0	JCK-1
.......................ggaucuuguucgacagca...	5	0	LT-2
.......................Ugaucuuguucgacagcacu...	1	1	LT-1
.......................ggaucuuguucgacagcacu...	1	0	JCK-2
.......................ggaucuuguucgacagcacu...	1	0	LT-2
.......................ggaucuuguucgacagcacu...	1	0	LT-1
.......................ggaucuuguucgacagcacu...	2	0	JCK-1
.......................ggaucuuguucgacagcacu...	5	0	JCK-3
.......................ggaucuuguucgacagcacuc..	2	0	JCK-3
.......................ggaucuuguucgacagcacuc..	3	0	JCK-2
.......................ggaucuuguucgacagcacuc..	1	0	LT-1
........................gaucuuguucgacagcacu...	2	0	JCK-1
........................gaucuuguucgacagcacu...	2	0	JCK-3
........................gaucuuguucgacagcacucc...	6	0	JCK-3
........................gaucuuguucgacagcacucc...	3	0	JCK-1
........................gaucuuguucgacagcacucc...	8	0	JCK-2
.........................aucuuguucgacagcacu...	1	0	LT-3
.........................aucuuguucgacagcacuc..	3	0	JCK-3
.........................aucuuguucgacagcacuc..	2	0	JCK-2
.........................aucuuguucgacagcacuc..	1	0	LT-3
.........................aucuuguucgacagcacuc..	2	0	JCK-1
.........................aucuuguucgCcagcacucc...	1	1	JCK-2
.........................aucuuguucgacagcacucc...	6	0	LT-1
.........................Cucuuguucgacagcacucc...	1	1	JCK-2
.........................aucuuguucgacagcacucc...	21	0	JCK-1
.........................aucuuguucgacagcacucc...	36	0	JCK-3
.........................aucuuguucgacagcacucc...	45	0	JCK-2
.........................aucuuguucgacagcacuccc..	17	0	JT-1
.........................aucuuguucgacagcacuccc..	98	0	JCK-3
.........................aucuuguucgacagcacuUcc..	1	1	JCK-1
.........................aucuuguucgacagcacuUcc..	1	1	JCK-2
.........................aucuuguucgacagcacuccc..	72	0	JCK-1
.........................aucuuguucgaAagcacuccc..	1	1	JCK-1
.........................aucuuguucgacagcacuccc..	109	0	JCK-2
.........................aucuCguucgacagcacuccc..	1	1	JCK-2
.........................Cucuuguucgacagcacuccc..	1	1	JCK-2
.........................aucuuguucgacagcacuccca...	1	0	JCK-2
..........................ucuuguucgacagcacuc..	2	0	LT-3
..........................ucuuguucgacagcacuc..	1	0	LT-1
..........................ucuuguucgacagcacucc...	4	0	JCK-3
..........................ucuuguucgacagcacucc...	5	0	JCK-2
..........................ucuuguucgacagcacucc...	1	0	JCK-1
..........................ucuuguucgacagcacuccc..	10	0	JCK-3
..........................ucuuguucgacagcacuccc..	18	0	JCK-2
..........................ucuuguucgacagcacuccc..	10	0	JCK-1
..........................ucuuguucgacagcCcuccc..	1	1	JCK-2
..........................ucuuguucgacagcacuccc..	1	0	LT-3
..........................ucuuguucgacagcacuccca...	2	0	JCK-2
..........................ucuuguucgacagcacuccca...	1	0	JCK-3
............................uuguucgacagcacucccauc...	2	0	JCK-2
....accggaagugcugucgagcgug..	2	0	LT-2
....Uggaagugcugucgagcgug..	4	1	JCK-2
....Uggaagugcugucgagcguga...	1	1	JCK-2
....aagugcugucgagcgugauA...	1	1	JCK-2
....aagugcugucgagcgugauuc..	1	0	LT-1
....aagugcugucgagcgugauucA...	5	1	LCK-3
....aagugcugucgagcgugauuccg..	4	0	LT-3
....aagugcugucgagcgugauuccg..	1	0	JCK-1
....aagugcugucgagcgugauuccga...	6	0	LT-2
....aagugcugucgagcgugauuccgaU..	1	1	JCK-3
....agugcugucgaAcgugau..	1	1	LCK-1
....gugcugucgagcgugauA...	2	1	JCK-3
....gugcugucgagcgugauu...	1	0	JCK-2
....gugcugucgagcgugauA...	1	1	LT-1
....gugcugucgagcgugauu...	2	0	LT-3
....gugcugucgagcgugauu...	4	0	JCK-1
....gugcugucgagcgugauu...	3	0	LT-1
....gugcugucgagcgugauuc..	3	0	JCK-1

Mature (uggaucuuguucgacagcacu) — Star (gugcugucgagcgugauuccg)

gggguguuguucauuuuacccucuggaucuuguucgacagcacucccaucaucuuuguucauguuugcgguuagggguguuauauauccacccccaaucucaaucucauaugugacaugcaccggaagugcugucgagcgugauuccgaaggugcuguucagcaacauacagauggaucgagcaacuacacugccugcuauauaugcccaucaucaucuucuacuuaaccuugcuuauuuauuuauuauuca

gugcugucgagcgugauuc	1	0	JCK-3
gugcugucgagcgugauuc	4	0	LCK-3
gugcugucgagcgugauuU	5	1	LT-2
gugcugucgagcgugauuc	3	0	JCK-2
gugcugucgagcgugauucc	2	0	JCK-3
gugcugucgagcgugauucc	7	0	JCK-2
gugcugucgagcgugauucc	2	0	LT-1
gugcugucgagcgugauucc	2	0	JCK-1
gugcugucgagcgugauucc	1	0	LT-3
gugcugucgagcgugauuccg	153	0	JCK-2
gugcugucgagcgugauuccU	1	1	JCK-1
gugcugucgagcgugauuccU	1	1	JCK-2
gugcugucgagcgugauuUcg	1	1	JCK-3
gugcugucgagcgugauuccg	10	0	LT-2
gugcugucgagcgugauuccg	11	0	LT-1
gugcugucgagcgugauuccg	105	0	JCK-3
gugcugucgagcgugauuccU	1	1	JCK-3
gugcugucgagcgugauuccg	82	0	JCK-1
gugcugucgagcgugauuccA	2	1	JCK-2
gugcugucgagcgugauuccg	16	0	LT-3
gugcugucgagcgugauuccga	8	0	LCK-3
gugcugucgagcgugauuccga	5	0	JCK-3
gugcugucgagcgugauuccga	11	0	JCK-1
gugcugucgagcgugauuccga	3	0	LT-1
gugcugucgagcgugauuccga	13	0	JCK-2
gugcugucgagcgugauuccga	2	0	LT-2
gugcugucgagcgugauuccgaU	3	1	LT-3
ugcugucgagcgugauuc	1	0	LT-1
ugcugucgagcgugauuU	1	1	LT-1
ugcugucgagcgugauucc	1	0	LT-3
ugcugucgagcgugauucc	2	0	JCK-3
ugcugucgagcgugauucc	1	0	LT-1
ugcugucgagcgugauuccg	15	0	JCK-3
ugcugucgagcgugauuccg	1	0	LT-2
ugcugucgagcgugauuccg	15	0	JT-1
ugcugucgagcgugauuccg	7	0	LT-3
ugcugucgagcgugauuccg	1	0	LT-1
ugcugucgagcgugauuccU	2	1	JCK-2
ugcugucgagcgugauuccg	26	0	JCK-2
ugcugucgagcgugauuccg	23	0	JCK-1
ugcugucgagcgugauuccga	7	0	JCK-1
ugcugucgagcgugauuccga	15	0	JCK-2
ugcugucgagcgugauuccga	11	0	JCK-3
ugcugucgagcgugauuccga	4	0	LT-3
ugcugucgagcgugauuccga	2	0	LT-1
ugcugucgagcgugauuccgaagg	3	0	JCK-1
ugcugucgagcgugauuccgaagg	1	0	LT-3
ugcugucgagcgugauuccgaagg	9	0	JCK-2
ugcugucgagcgugauuccgaagg	3	0	JCK-3
ugcugucgagcgugauuccgaaggu	1	0	JCK-3
ugcugucgagcgugauuccgaaggu	2	0	JCK-2
ugcugucgagcgugauuccgaagguU	4	1	JT-1
cugucgagcgugauuccga	8	0	LCK-3
caucaucaucAucuacuua	1	1	JCK-1
